# Radio-Absorbing Magnetic Polymer Composites Based on Spinel Ferrites: A Review

**DOI:** 10.3390/polym16071003

**Published:** 2024-04-06

**Authors:** Vladimir G. Kostishin, Igor M. Isaev, Dmitrij V. Salogub

**Affiliations:** Department of Materials Technology of Electronics, National Research University of Technology “MISA”, Leninsky Prospect, 4, 119049 Moscow, Russia; isa@misis.ru (I.M.I.); salogub.dmitry@yandex.ru (D.V.S.)

**Keywords:** magnetic polymer composites, radar absorbing materials, shielding materials, spinel ferrite, electromagnetic radiation, magnetic permeability, dielectric permeability, reflection spectrum of the sample on the metal plate

## Abstract

Ferrite-containing polymer composites are of great interest for the development of radar-absorbing and -shielding materials (RAMs and RSMs). The main objective of RAM and RSM development is to achieve a combination of efficient electromagnetic wave (EMW) absorption methods with advantageous technological and mechanical properties as well as acceptable weight and dimensions in the final product. This work deals with composite RAMs and RSMs containing spinel-structured ferrites. These materials are chosen since they can act as efficient RAMs in the form of ceramic plates and as fillers for radar-absorbing polymer composites (RAC) for electromagnetic radiation (EMR). Combining ferrites with conducting fillers can broaden the working frequency range of composite RAMs due to the activation of various absorption mechanisms. Ferrite-containing composites are the most efficient materials that can be used as the working media of RAMs and RSMs due to a combination of excellent dielectric and magnetic properties of ferrites. This work contains a brief review of the main theoretical standpoints on EMR interaction with materials, a comparison between the radar absorption properties of ferrites and ferrite–polymer composites and analysis of some phenomenological aspects of the radar absorption mechanisms in those composites.

## 1. Introduction

The use of electromagnetic radiation (EMR) in science and technology provided a wide range of opportunities and triggered intense technical progress in the 20th century. However, the benefits of EMR have a reverse side, electromagnetic pollution. This is commonly treated as the growing electromagnetic wave (EMW) intensity in urban spaces, accommodation areas, industrial zones and even in the whole environment within a wide EMR spectrum, from radio frequencies to microwaves, excluding ionizing EMR [1,2,3,4,5]. One of the most widely discussed problems is the long-term human body exposure to non-ionizing low-power EMR [6,7,8,9]. For example, there are indications that a number of borderline personality disorders, depression, hyperkinetic behavior syndrome, child hyperactivity and suicidal tendencies can originate from long-term EMR exposure in everyday activity [10]. Typical EMR sources surrounding humans in the 21st century are mobile phones, microwave communication devices, TV sets, Wi-Fi, computers, antennas, satellite and mobile communication. Those EMR sources can emit in a range from extremely low frequencies to microwaves (~1 Hz to hundreds of GHz). Microwave radiation doubtlessly exerts a direct impact on the human body by interacting with the water molecules inside it. This interaction causes sleeping disorders, emotional instability and possible cumulative effects, leading to carcinoma formation. There are numerous studies demonstrating the effect of the habitual things surrounding us on human health. Furthermore, electromagnetic pollution is a serious challenge in the design of electronic devices, antennas and other EMW equipment [11,12]. Parasitic signals, noise and high electromagnetic background can disrupt equipment operation. To avoid the development of critical equipment operation conditions, one should undertake measures providing electromagnetic compatibility between different equipment units or maximizing equipment performance under specific operation conditions. The above list of problems originating from the wide spreading of EMR is not exhaustive, but those problems are sufficient to demonstrate the necessity of reducing the electromagnetic background in living areas. The best solution to minimizing EMW distribution in a limited space is the use of electromagnetic shields (EMSs), the simplest of which are metallic sheets (plates) or wire mesh. However, when this solution is used, EMW multiple reflection causes only electromagnetic background redistribution in the space and can even aggravate the problem [13]. Minimization of the electromagnetic background requires the use of radar-absorbing materials (RAMs), capable of complete internal absorption of EMW energy and converting it to heat. One can, thus, avoid EMW multiple reflection and reduce electromagnetic background in a large space. Advanced civilian RAM should meet the following requirements: light weight (low density), easy mechanical treatment, manufacturability (production route should not include complex processes), maximally wide working frequency range and atmospheric resistance [14]. Good options are polymer composites, in which organic materials act as the matrix and EMR-interacting powders as the fillers [15]. Logically, the performance of radar-absorbing composites is largely determined by the choice of fillers readily interacting with EMR. The fillers lead to dielectric and magnetic losses, which will be dealt with below. Polymers are typically considered only as matrices to bind the other components, because most of the currently available and widely used thermoplastic and thermosetting composites do not exhibit radar-absorbing properties in the NHz and GHz EMR spectrum regions that are most widely used in civilian applications [16]. One cannot, however, definitively affirm that the dielectric properties of polymers do not affect radar absorption by polymer composites [17]. Later, we will demonstrate that changes in the dielectric and magnetic permeabilities, even by a few fractions of a unit, can cause noticeable changes in the radar absorption properties of radar-absorbing composites.

## 2. EMR Interaction with Materials

Before addressing the radar-absorbing properties of RAM, one should consider the main definitions relating to the electrophysical parameters of materials and theoretical standpoints on EMW interaction with materials. It should be stressed that EMR is defined as the propagation of interrelated electric and magnetic fields through tremendous distances in space. EMR interacts both with the electric charges and the magnetic moments of the material through which it propagates. An electric field applied to a material shifts opposite charges or rotates electric dipoles in the material bulk. This phenomenon is also referred to as polarization. One can indirectly assess the polarization capability of a material from the change in its relative dielectric permeability *ε_r_*, which is contained in one of Maxwell’s constitutive equations. Since EMR propagation is described by a harmonic sine law, the oscillatory movement of charges occurs. Since the harmonic processes are written in complex numbers, the dielectric permeability is written as follows [18]

(1) $${\varepsilon_{r}}^{*}={\varepsilon_{r}}^{\prime }+i\varepsilon_{r}\prime \prime$$

The real part of ε_r_* is related to polarization processes and characterizes the material’s capability to accumulate charge, whereas the imaginary part is related to polarization (dielectric) losses. Charges can be covalent electrons, electrons and holes in the conduction band (or in the valence band), polarons, dipoles and defect-related electrons. High-relative dielectric permeability is inherent to ferroelectrics and ionic bond crystals. Dielectric permeability is usually measured in an AC electric field (up to 1 MHz) using EMR in the microwave region [19]. Furthermore, application of an AC electric field produces bias, which also depends on polarization processes and the DC electrical conductivity of the material.

Magnetic field application to a material produces magnetic induction through reorientation of the magnetic moments of atoms or ions. The magnitude of magnetic induction is proportional to the magnetic permeability of the material. For EMR interaction with materials, the magnetic permeability is written in a complex form:

(2) $${\mu_{r}}^{*}={\mu_{r}}^{\prime }+i\mu_{r}\prime \prime$$

By analogy with complex dielectric permeability, the real part of complex magnetic permeability shows the material’s magnetization capability, whereas the imaginary part characterizes the quantity of energy lost for remagnetization. The complex dielectric permeability ε_r_* and the complex magnetic permeability μ_r_* exhibit a clear frequency dependence (frequency dispersion). In general, this originates from the fact that the electric dipoles and magnetic moments of atoms exhibit a delay relative to the rapidly changing electric and magnetic fields. To characterize the properties of radar-absorbing materials, one should know the frequency dependences of the complex dielectric permeability and the complex magnetic permeability. To demonstrate this law, one can consider EMW attenuation in a material with non-zero loss. The radar absorption phenomenon is related to loss of the energy in a flat EMW during its propagation through the bulk of a material and conversion to heat. This process is accompanied by a decrease in the amplitude of the electromagnetic wave. This decrease is written as $I\left(x\right)=I_{0}\cdot e^{-\alpha \cdot x}$, where α is the attenuation coefficient. The attenuation coefficient is calculated using the following formula:

(3) $$\alpha =\frac{\sqrt{2\pi f}}{c}\sqrt{\mu_{r}^{\prime \prime }\varepsilon_{r}^{\prime \prime }-\mu_{r}^{\prime }\varepsilon_{r}^{\prime }+\sqrt{{\left(\mu_{r}^{\prime \prime }\varepsilon_{r}^{\prime \prime }-\mu_{r}^{\prime }\varepsilon_{r}^{\prime }\right)}^{2}+{\left(\mu_{r}^{\prime \prime }\varepsilon_{r}^{\prime }+\mu_{r}^{\prime }\varepsilon_{r}^{\prime \prime }\right)}^{2}}}.$$

One can, therefore, demonstrate in a first approximation that radar absorption in a material depends on its magnetic permeability and dielectric permeability, as well as on the frequency dependences. There are literary indications that EMR interaction with monolithic materials can occur through several possible EMW interaction scenarios: EMW reflection from the material’s surface, EMW absorption by the material and propagation through the material. Three relative coefficients are often used for EMW power or amplitude characterization. The sum of these coefficients equals 1. These coefficients are the reflection coefficient (*R*), the absorption coefficient (*A*) and the transmission coefficient (*T*) [20]:

(4) A+T+R=1.

It is obvious that radar-shielding materials (RSMs) should exhibit the highest reflection (or absorption) coefficient and the lowest transmission coefficient, whereas RAMs should have the highest absorption coefficient and the lowest reflection and transmission coefficients. One should note, however, that RAM applicability criteria may vary depending on RAM testing setup. For example, test RAMs are often placed on a perfect reflector (metal) plate. Then, the measurement criterion is the reflection coefficient of the metallic plate (reflection loss). The reflection coefficient is calculated using the formulas given below [19]:

(5) $$Z_{in}=Z_{0}{\left(\frac{{\mu_{r}}^{*}}{{\varepsilon_{r}}^{*}}\right)}^{\frac{1}{2}}\tanh\left[i\left(\frac{2\pi fh}{c}\right){{(\mu_{r}}^{*}{\varepsilon_{r}}^{*})}^{\frac{1}{2}}\right]$$

(6) $$R_{l}=20log\left|\frac{Z_{in}-Z_{0}}{Z_{in}+Z_{0}}\right|$$

where *Z_in_* is the wave impedance of the specimen, *Z*_0_ is the characteristic impedance of free space, h is the absorber thickness and c is the light velocity.

These formulas describe experimental data very well and allow one to evaluate the radar absorption of composites from experimental spectra of complex ε_r_* and μ_r_* without the need to produce massive and costly specimens. The lowest *R*_1_ can be achieved by matching the impedances *Z_in_* and *Z*_0_. All the aspects relating to the effect of RAM thickness and electrophysical parameters on the interference (resonance) absorption were addressed in good detail earlier [21]. There are also methods for calculating the matching conditions, which, depending on EMR frequency (wavelength), can yield sets of real and imaginary parts of ε_r_* and μ_r_* or thicknesses for which EMR will be attenuated to the greatest extent at the preset abovementioned parameters [22,23].

**Figure 1:**
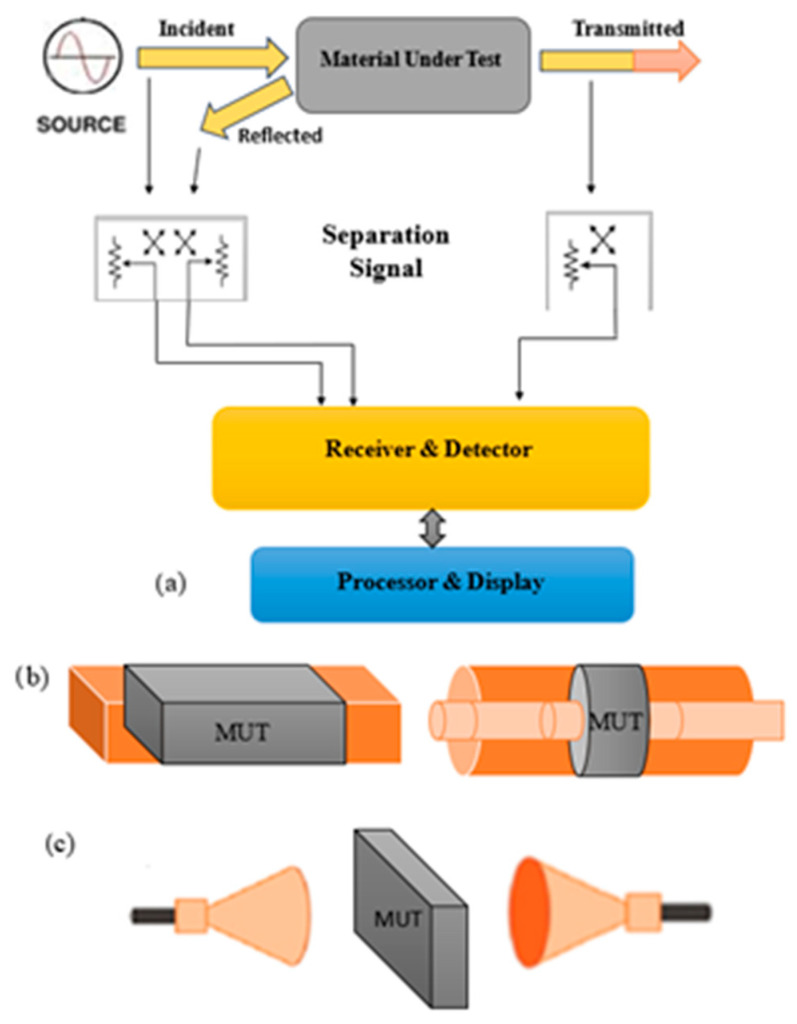
(**a**) Basic diagram of electromagnetic parameter measurement using a vector network analyzer and (**b**) typical RAM and (**c**) RSM measurement setups.

When it comes to measuring the radar-shielding properties of RSMs, the experiments are conducted without a metallic plate behind the RSM. Both measurement setups can be easily implemented with a vector network analyzer, which allows for measuring *A*, *T* and *R* (or the S parameters) as well as the EMW phase over a wide frequency range. This set of measured parameters allows for calculating the frequency dependence of the complex dielectric and magnetic permeabilities using various methods [24,25]. The most widely used options are a coaxial waveguide, a rectangular waveguide and free-space measurements (Figure 1).

Shielding effectiveness (SE) is often distinguished out of the parameters describing the radar-shielding properties of materials. The complete shielding effectiveness (or the transmission coefficient) can be written as *SE_T_ =* 20*lg(E_t_/E*_0_*) =* 20*lg(H_t_/H*_0_*)* through the magnitudes of the electric and magnetic fields. At *SE_T_* > 10 dB, the shielding effectiveness contains two terms: reflection shielding *SE_R_* and absorption shielding *SE_A_* [26]:

(7) $${SE}_{T}={SE}_{R}+{SE}_{A},$$

which are written as

(8) $${SE}_{R}=10lg\left(1-R\right),$$

(9) $${SE}_{A}=10lg\left(\frac{T}{1-R}\right)=8686\;h\alpha ,$$

where *α* is the attenuation coefficient; *h* is the sample thickness.

At the end of this section, it should be noted that magnetoelectric sensors, which are very well described in a review [27], can be successfully used to detect electromagnetic pollution.

## 3. Electromagnetic Properties and Synthesis Methods of Spinel Ferrites

**Figure 2:**
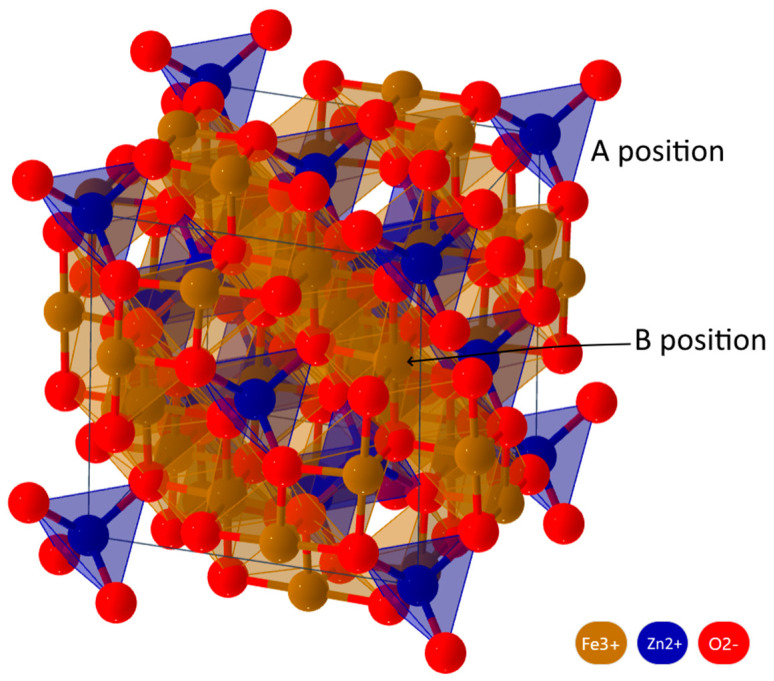
Crystal structure of zinc spinel ferrite.

Among transition metal oxides, one can distinguish iron oxides, iron co-oxides with other metal oxides and the solid solutions of oxides. The abovementioned iron co-oxides with other metal oxides are commonly referred to as ferrites [28]. Out of the variety of ferrites, one can separate spinel ferrites, which are widely used as magnetic materials in electrical engineering, electronics and microwave devices [29]. The term spinel ferrite originates from the fact that the crystallographic structure of this material is isomorphic to that of spinel crystals (MgAl_2_O_4_), having the Fd-3m space group. The crystal lattice of this ferrite is shown in Figure 2. There are normal spinel ferrites, mixed spinel ferrites and converted spinel ferrites [30]. In the latter (converted) spinel ferrites, Me^2+^ and Fe^3+^ cations occupy the octahedral B positions, whereas the tetrahedral A positions are occupied by Fe^3+^ iron cations in a normal spinel; Me^2+^ cations occupy only the A positions, whereas Fe^3+^ cations occupy the octahedral B positions. The example shown in Figure 2 is normal spinel ZnFe_2_O_4_, where Zn^2+^ ions occupy only the tetrahedral positions (the blue polyhedrons), whereas Fe^3+^ ions occupy the octahedral positions (the green polyhedron). In mixed spinel ferrites, 3+ and 2+ valence cations occupy both sublattices. The general formula of spinel ferrites can be written as MeFe_2_O_4_, where Me is a bivalent metal. Ferrite solid solutions are used the most widely, e.g., Ni-Zn, Mn-Zn, Mg-Zn and Co-Zn spinel ferrites. Detailed analysis of the crystallographic structure of ferrites is required for understanding the origin of their magnetic properties. It is considered that the magnetic properties originate mainly from the super-exchange interaction of 3d shell iron electron spins in the A and B sublattices [31]. As a result, the magnetic moments of the two sublattices arrange in an antiparallel manner, thus enabling ferromagnetic ordering in spinel ferrites. The arrangement of cations in the sublattices and the configuration of their external electron shells determine the crystallographic magnetic anisotropy, the magnetic moment of the unit cell (or the saturation magnetization), the coercive force and the magnetic permeability of ferrites [32].

Changing the chemical composition of ferrites and, to an extent, avoiding the distortion of the spinel crystal cell, one can significantly affect the magnetic or electric properties of ferrite ceramics or powders. Ferrites have less expressed magnetic properties than iron alloys, but their low electrical conductivity and high chemical stability can be advantageous, e.g., in the production of RAC. Spinel ferrites are magnetically soft materials (low coercive force *H_c_* and high initial magnetic permeability μ_0_) with moderate anisotropic constant and ferromagnetic resonance frequency [28]. One can also note the relatively high Curie temperatures (the ferromagnetic to paramagnetic transition points) in the 100–300 °C range. In the absence of an external field, if the crystallographic anisotropy field acts as a magnetizing field, ferrite interaction with EMR produces the natural ferromagnetic resonance (NFMR) [33]. This phenomenon occurs if the EMW frequency coincides with the magnetic moment precession frequency in ferrite sublattices. The formula of ferromagnetic resonance as a function of an effective ferromagnetic field is often used in the literature [34]:

(10) $$\Omega_{res}=\gamma \cdot H_{eff},$$

where *H_eff_ = H_a_ + H_d.f._ + H_g_ + H_σ_*, *H_a_* is the crystallographic magnetic anisotropy field, *H_d.f._* is the field of demagnetizing factors, *H_g_* is the growth anisotropy field, *H_σ_* is the stress anisotropy field and *γ* is the gyromagnetic ratio.

The ferromagnetic resonance frequency in magnetic polymer composites depends on intrinsic factors, e.g., magnetic particle shape, distribution pattern and concentration. Those factors can strongly affect the radar absorption properties of spinel ferrite-based RAC.

As a result of NFMR processes in spinel ferrites, the cutoff frequency at which the imaginary part of magnetic permeability grows rapidly and the real part of the magnetic permeability decreases are in the MHz region due to a low anisotropy field. However, efficient GHz spinel ferrite RAC can be produced, as shown below.

One should also dwell upon the main spinel ferrite production methods used by researchers and industry engineers. The most widely used spinel ferrite production method is ceramic technology (or the solid-state reaction method), for which compressed powdered oxides and carbonates with metal content as per the synthesized ferrite composition are sintered at high temperatures [31,35]. This method is used for the mass production of Ni-Zn and Mn-Zn ferrites in the form of bulk ceramics, but it has a number of disadvantages. The synthesis of high-density ceramics requires high temperatures and a long sintering time in resistive furnaces, while the magnetic parameters of the final products can vary substantially in one batch. To reduce the energy consumption of the process and increase the yield, one can use other annealing methods or sintering modifiers: radiation thermal sintering [36], reactive instantaneous sintering [37], spark plasma sintering [38], sintering using microwaves [39] and low-melting-point additions for sintering temperature reduction [40]. To use ferrite ceramics for RAC production, one should mechanically grind the as-sintered ceramic materials in mills to the required particle size.

To directly synthesize ferrites in the form of powdered fillers, one should use chemical solution synthesis methods. Oxides in those methods are replaced with metal salts or metal–organic compounds that are dissolved in water or other solvents. The general principle of those methods is to mix the metal salt solution with polymers, alkali and other reactants to obtain the precursor in the form of a residue. The precursor should then be heat treated to obtain the spinel ferrite phase. The simplest ferrite powder synthesis method is co-precipitation, for which the residue from the solution is dried, filtered and annealed in a furnace [41]. One can distinguish this from the hydrothermal (solve-thermal) method for which the salt solution with a mineralizer is held in an autoclave at relatively low temperatures (180–500 °C) for obtaining ferrite nanoparticles [42,43]. The sol–gel method is also widely used, for which a hydrolysis reaction between salts, catalysts and gel-forming agents produces 3D structures with a homogeneous metal cation distribution. Heat treatment converts the solution from the sol condition to the gel one, and a further increase in temperature produces ferrite nanoparticles [44]. A gel self-inflammation reaction agent can be added to sol. The flash point is sufficient for the formation of the ferrite phase in the final product. The microemulsion [45], ultrasonication [46] and mechanical activation [47] methods are also used.

## 4. Radar-Absorbing Parameters of Ferrites and Ferrite–Polymer Composites

Analysis of publications over the most recent 20 years suggests that the main trends in the research and production of ferrite–polymer composites are the transition to the submicron or nanometer scales and the development of and improvement in new and existing ferrite filler production methods. The nanometer scale transition implies the use of ferrite particles with smaller than 100 nm sizes in one of the directions or combinations of ferrite particles with other nanoparticles [48,49]. However, insufficient attention is paid to the problem of particle agglomeration during composite synthesis, which offsets the advantages of their small size. The main concept of ferrite–polymer composite synthesis is to fill the polymer matrix with distributed filler particles. Some materials can be chemically bound to hybrid composite fillers. A special example is the technology for which the polymer is used as a conducting shell in “magnetic core (ferrite)–conducting shell (polymer)” structures [50]. Importantly, the use of such hybrid composite fillers solves the problem of the homogeneous distribution of magnetic fillers in the RAC bulk. Detailed analysis of RAC compositions reported so far also provides examples of ferrite–polymer composites with additional dielectric (ferroelectric) fillers [23], conductive fillers [51], combinations of conductive and dielectric fillers and combinations of magnetically soft and magnetically hard fillers [34]. It is commonly believed that the use of fillers having different magnetic and electric properties increases the overall electromagnetic energy loss in composites due to a combination of different absorption mechanisms and broadens the working range of the RAC.

First of all, one should demonstrate that spinel ferrites in the form of continuous ceramics have good radar-absorbing properties in the RF range. Good examples are Ni-Zn, Mn-Zn and Li-Mn-Zn ferrites, which are solid solutions of the simple spinels NiFe_2_O_4_, ZnFe_2_O_4_, MnFe_2_O_4_ and Li_0.5_Mn_0.5_Fe_2_O_4_. Due to the complex microstructure of ferrite ceramics (single-crystal grains and amorphous grain boundaries), ferrites exhibit intense interfacial polarization at low EMR frequencies. As a result, ferrites demonstrate tremendous *ε*’*_r_* and high dielectric losses [52,53]. The initial magnetic permeability of magnetically soft spinel ferrites (ceramics) can vary over a wide range, from several decades to 25,000. The high magnetic and dielectric permeabilities provide the excellent radar absorption properties in the low-frequency EMR region. This can be traced by analyzing the formula which qualitatively illustrates the RAM thickness at which interference and maximum radar absorption occur, as a function of the dielectric and magnetic permeabilities.

(11) $$h=\frac{nc}{4f\sqrt{\varepsilon_{r}^{*}\mu_{r}^{*}}},$$

where *c* is the light velocity, *f* is the frequency and *n* an odd number 1, 3, 5 … etc.…

It follows from Equation (11) that high *R_l_* absolute values in spinel ferrites will be observed at low frequencies and a constant absorber material thickness. Ni_0.39_Zn_0.61_Fe_2_O_4_ ceramic with Bi_2_O_3_ addition and a test specimen thickness *h* = 7 mm exhibited high EMR attenuation to −30 dB (the *R_l_*(*f*) peak is at 200 MHz) and a 980 MHz absorption bandwidth at −10 dB [54]. The authors attributed the expressed radar-absorbing properties to magnetic loss for natural ferromagnetic resonance and domain wall resonance (DWR). Our laboratory conducted a study of the radar-absorbing properties of 400НН, 1000НН, 2000НН (Ni-Zn ferrites), 2000НМ (Mn-Zn ferrites) and Li-Mn-Zn ferrites synthesized in Russia. Ni-Zn ferrites had absorption peaks in the reflection spectrum of the sample on the metal plate in the range of–(18–13) dB. The peak positions were in the frequency range 200–700 MHz, and the absorption bandwidth was Δ*f*(−10 dB) = 1050–1300 MHz. The RAM thickness was 6 mm, the dielectric permeability *ε*′*_r_* was within 10 and the magnetic permeability *μ*′*_r_* in the sub-resonance region was 500–1000. It was found that the Ni-Zn also has predominantly magnetic loss, with a small fraction of dielectric loss due to hopping polarization [55]. These losses occur in defective ionic bond crystals, which is the case for ferrites with oxygen deficiency after annealing [56]. By and large, since Ni-Zn is quite a good dielectric, it does not exhibit large dielectric loss in the MHz and GHz regions. The same is true for the Li-Mn-Zn ferrites, but better impedance-matching conditions are provided for the minimum *R_l_* of −22 dB at a higher frequency than for the Ni-Zn ferrite (1.34 GHz) and a 2 GHz absorption bandwidth [57]. The good radar absorption properties were also observed for the Li-Zn ferrites with CuO and MgO additions. For 6–10 mm thicknesses, the maximum *|R_l_|* was within 25–48 dB, the peak position being at 0.2–0.8 GHz [58].

The situation is different for Mn-Zn ferrites synthesized by sintering in an Ar gas atmosphere: the electrical resistivity of the ferrites was close to that of semiconductors at sufficiently high magnetization. This resulted in the cutoff frequency *μ_r_*′(*f*) lying in a range of several MHz with high (~30) dielectric permeability being retained up to the GHz EMR region. The high conductivity of the grains in the Mn-Zn spinel is caused by the presence of [Fe^2+^-Fe^3+^] ions or [Me^2+^-Fe^3+^] complexes in the octahedral positions. Electrical conductivity between the ions and the ion complexes occurs via a hopping mechanism (electrons jump between energy bands due to electron–phonon interaction) [59]. The Mn-Zn ferrites can contain Mn and Fe ions with different valences, with the oxidation degree for Mn being from 2+ to 4+. Thus, Mn-Zn ferrites with a spinel structure have low electrical resistivity and expressed magnetic properties. These electrophysical properties of Mn_1−x_Zn_x_Fe_2_O_4_ determine the radar absorption properties of the ceramic specimens. Our experiments showed that for the setup with a perfect reflector, an impedance mismatch causes the *R_l_* coefficient in the 1–100 MHz range to be at least −10 dB for a thickness of 5–9 mm. Noteworthily, the peak reflection coefficients were at 1–10 MHz, making the MN-Zn ferrite a potentially effective low-frequency radar absorbent.

Regarding the radar-screening properties of the abovementioned ferrites, one should point out that the absence of expressed dielectric loss in the Ni-Zn and Li-Mn-Zn ferrites precludes their use as an RSM in the MHz and GHz ranges at thicknesses of 5–10 mm. The shielding effectiveness *SE_T_* of the Ni-Zn and Li-Mn-Zn ferrites is within 10 dB. In the MN-Zn ferrite, eddy current loss caused by high conductivity increases dielectric and magnetic losses, allowing one to achieve *SE_T_* of 10 to 20 dB in the 1–7 GHz range for 5 mm thick material.

The situation for ferrite–polymer composites is completely different. Our department conducted studies of different ferrite–polymer composite compositions (both two-component and multicomponent ones) in which thermoplastic polymers, such as polyvinylidenfluoride (PVDF), polyvinyl alcohol (PVA) and polystyrene, were used as polymer matrices. The main change in the radar-absorbing properties of the RAC with spinel ferrite fillers in comparison with those of the initial ferrites was a shift in the absorption peak in the *R_l_*(*f*) spectra towards higher frequencies with a decrease in the ferrite concentration. At a ferrite filler weight fraction of 80–20% for the Ni-Zn and Mn-Zn ferrites, the absorption peaks shift from the MHz range to the GHz one. Furthermore, the resonance frequency in the μ_r_*(*f*) spectrum also shifts towards higher frequencies with a decrease in the ferrite concentration. Earlier, changes in the μ_r_*(*f*) frequency position for ferrite polymer composites were studied by Tsutaoka [60,61,62,63]. Tsutaoka also found that the NFMR and DWR frequencies shift towards higher frequencies with a decrease in the ferrite concentration. We also observed, in our works, a similar behavior of resonance frequencies with changes in the ferrite concentration. It was found that the NFMR frequency shift is greater than the DWR frequency shift, and the imaginary parts of the magnetic susceptibility *χ*″ which are related to NFMR are always higher at maximum absorption frequencies [64,65]. We, therefore, assumed that NFMR makes the greatest contribution to radar absorption. This can be accounted for by the change in the effective field *H_eff_* in the composite due to the demagnetization factor [23]. On the other hand, this can be qualitatively interpreted using Snoek’s law [66]:

(12) $${(\mu }_{0}-1)f_{r}^{2}={\left(\gamma 4\pi M_{s}\right)}^{2}$$

where *f_r_* is the resonance frequency (cutoff frequency) and *M_s_* is the saturation magnetization of ferrite; $\mu_{0}$ is the magnetic constant.

The magnetization of ferrite–polymer composites (relative to ferrite weight) does not change, whereas the magnetic permeability *μ*_0_ decreases considerably due to a decrease in the magnetic flow in a non-continuous magnetic medium [66]. Then, in order for the equation to hold, *f_r_* should increase, which is observed in the experimental ferrite–polymer composite spectra.

**Figure 3:**
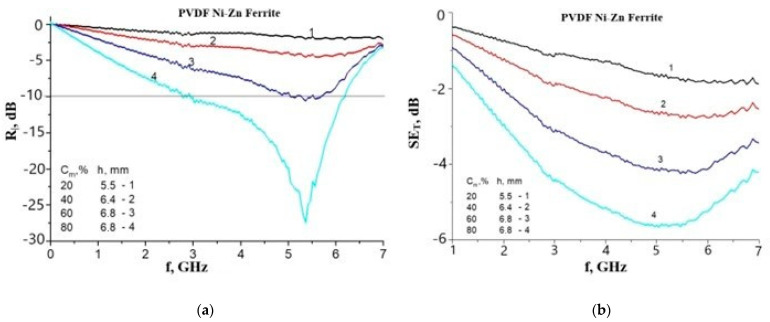

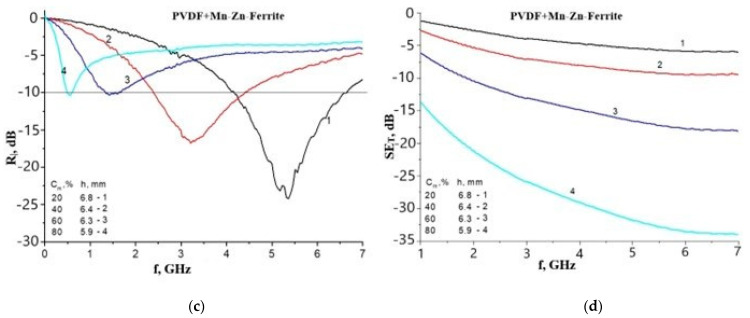
Illustration of the effect of filler electrical properties on the radar absorption properties of ferrite–polymer composites with Mn-Zn and Ni-Zn ferrite fillers. The results were obtained at NUST MISiS by the Department of Electronics Materials Technology. (**a**) *R_l_*(*f*) spectrum of PVDF/Ni-Zn ferrite composite, (**b**) *SE_T_*(*f*) spectrum of PVDF/Ni-Zn ferrite composite, (**c**) *R_l_*(*f*) spectrum of PVDF/Mn-Zn ferrite composite, (**d**) *SE_T_*(*f*) spectrum of PVDF/Mn-Zn ferrite composite.

We found excellent radar absorption properties of two-component RACs with Ni-Zn and Li-Mn-Zn spinel ferrites at weight fractions of 60–80% (volume concentrations of 25–61%) with *R_l_* = −33.8–−20 dB and an absorption bandwidth of Δ*f* (−10 dB) in the 2–4 GHz range and at 2–7 GHz frequencies [18,55,67]. The matrices were PVDF, PVA and polystyrene. Those parameters can be attributed to high magnetic losses in the composite (the NFMR and DWR losses) and impedance-matching conditions. However, even better parameters were observed for composites having the same matrices with Mn-Zn ferrite inclusions. This is well illustrated in Figure 3.

It was stressed in earlier publications that the use of sole Ni-Zn ferrite (especially nanoparticles synthesized using the co-precipitation, hydrothermal and sol–gel methods) with a high electrical resistivity as a filler can impose restrictions on device weight and dimensions due to the absence of expressed dielectric losses and eddy current loss in the GHz range [68,69,70,71]. However, excellent results [72,73] can be obtained for multicomponent spinel ferrite solid solutions with high ferrite filler concentrations. For example, the NiCuZn/ferrite composite with La substitution (Ni_0.35_Co_0.15_Zn_0.5_La_x_Fe_2−x_O_4_ (x = 0 − 0.06))/paraffin (in a 6:1 ratio) with a thickness of 4 mm and x = 0.02 had a peak absorption of −34 dB at 5.5 GHz (absorption bandwidth 5.5 GHz). From this viewpoint, good results were obtained for MN-Zn ferrite fillers with lower electrical resistivity than for Ni-Zn, Mg-Zn, Co-Zn and Li ferrites. For a 20–40 wt. % filler content (9–30 vol.%), the RAC with Mn-Zn ferrite had *|R_l_|* in the 20–44 dB range at 2–7 GHz and a bandwidth of ~2 GHz at −10 dB (for a 5–7 mm thickness). For higher filler contents, the impedance-matching condition is not met, and, hence, the maximum attenuation (dB) is lower. Similar results were obtained for the rubber/Mn-Zn ferrite composite [74]. High-concentration thermoplastic/Mn-Zn ferrite composites (filler content, 60–80 wt. %) can be considered as radar-shielding materials with *SE_T_* in the −33–−15 dB range at a reflection coefficient *SE_R_* = −3 dB. The radar-shielding properties of composites with Mn-Zn ferrites are caused by the small skin layer, which, in turn, is caused by a high attenuation coefficient (Equation (3)). The high dielectric and magnetic permeabilities of those composites originate from electrical and magnetic percolation [23,75]. One can, therefore, state that even two-component ferrite–polymer composites can be used as efficient RAMs in the GHz EMR range.

The radar absorption properties can be improved by adding conductive components to ferrite–polymer RACs [76,77]. Co_0.2_Ni_0.4_Zn_0.4_Fe_2_O_4_/graphene composites in 5:1, 10:1, 15:1 and 20:1 ratios were obtained using joint hydrothermal synthesis (one-pot route) [78]. The authors noted that the Co-Ni-Zn ferrite nanoparticles were wrapped in graphene sheets due to electrostatic interaction. Strong interaction between carbon derivatives and spinel ferrite nanoparticles was also observed by other researchers [79,80]. Then, the synthesized Co_0.2_Ni_0.4_Zn_0.4_Fe_2_O_4_/graphene powders were mixed with paraffin to produce composite rings, measuring the radar absorption properties. A comparison between composites containing sole Co-Ni-Zn ferrite as a filler and the Co_0.2_Ni_0.4_Zn_0.4_Fe_2_O_4_/graphene ones yielded ε_r_*(*f*) spectra, which suggested higher real and imaginary parts of the dielectric permeability due to interphase polarization and higher dielectric loss. It is noteworthy that with an increase in the graphene concentration in the specimen, the maximum *|R_l_|* increases initially and then decreases. At excessive conductive graphene concentrations, the impedances are no longer matched, and strong EMR reflection from the RAC surface is observed. For 3 mm thick composites without graphene, *|R_l_|* was within 5.8 dB. With an increase in the graphene concentration, this parameter increased to 12, 15 and 31.3 dB and decreased to 16 dB for the same specimen thickness and concentration ratios of 20:1, 15:1, 10:1 and 5:1, respectively. In another work, MnFe_2_O_4_ and ZnFe_2_O_4_ were used as magnetic additions and multi-walled carbon nanotubes as the conductive filler [81]. The matrix was paraffin. The specimens were synthesized by mechanical mixing of all the components in the form of powders (total weight fraction of fillers, 40%). The specimens with a sole ferrite filler and multi-walled carbon nanotubes had a radar absorption coefficient of within 30 dB in the 8-12 GHz range, which is a sufficiently good result. However, the introduction of carbon nanotubes produces strong resonance peaks with the highest *|R_l_|* = 35–58 dB. The working frequency bandwidth was 4 GHz or greater for all the specimens synthesized. There are many literary data on the use of carbon-containing materials as RAC performance-improving additives [82,83].

**Figure 4:**
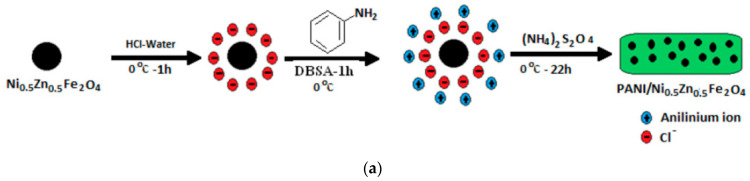

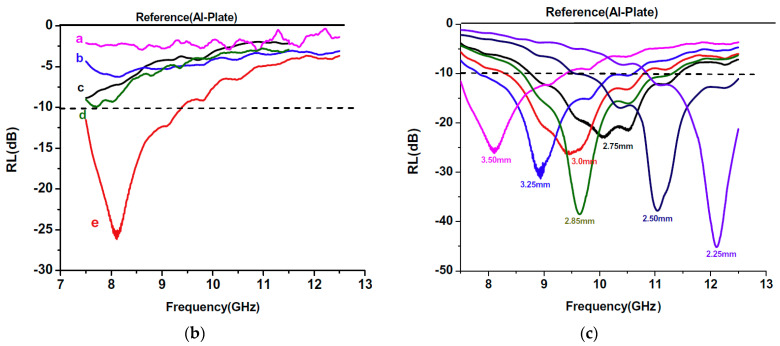
(**a**) Schematic of polyaniline/Ni-Zn ferrite synthesis setup, (**b**) *R_l_*(*f*) spectra of paraffin/Ni-Zn ferrite/polyaniline composites with different polyaniline/Ni-Zn ferrite ratios (legend: (a) Ni-Zn ferrite, (b) polyaniline/Ni-Zn ferrite = 2:1, (c) polyaniline/Ni-Zn ferrite = 1:2, (d) polyaniline, (e) polyaniline/Ni-Zn ferrite = 1:1) and (**c**) *R_l_*(*f*) spectra of paraffin/Ni-Zn ferrite/polyaniline composites for 1:1 polyaniline/Ni-Zn ferrite ratio and different RAC thicknesses.

A very efficient materials science solution for composites is to produce magnetic core/conductive shell structures. They are produced from spinel ferrite nanoparticles synthesized using the sol–gel or hydrothermal methods and monomers of conductive polymers, e.g., polyaniline or polypyrrole. A solution containing those components is added with a polymerization agent, with the nanoparticle/solution interface acting as the polymerization center. Illustrative results were obtained for polyaniline/Ni-Zn ferrite composites [50]. Figure 4 shows a schematic of a magnetic core/conductive shell composite synthesis setup and a comparison between the radar absorption parameters of the composites. It can be seen that the best radar absorption conditions are for a polyaniline/Ni-Zn ferrite ratio of 1:1; varying the thickness from 2.25 to 3.5 mm, one can cover the entire 8–12 GHz range. Similar structures were also studied earlier [84,85,86]. Magnetic core/conductive shell structures allow for controlling the frequency behavior of complex ε_r_* and μ_r_*, eventually making great changes to the radar absorption properties, e.g., resonance peak position and working bandwidth [87].

Excellent radar absorption properties can be obtained using ferroelectric polymers, i.e., PVDF and its copolymers [88]. For example, PVDF/nano-Mn_0.8_Zn_0.2_Cu_0.2_Fe_1.8_O_4_ ferrite films ~0.2 mm in thickness exhibit excellent radar absorption properties in the 12–18 GHz range, with a 6 GHz working bandwidth and −32 dB peak attenuation [89].

In order to increase the maximum operation frequencies and possibly the working bandwidth, attempts were made to synthesize RACs with fillers consisting of sintered magnetically soft and magnetically hard ferrite particles. Since magnetically hard ferrites have high magnetic crystallographic anisotropy fields, according to Equation (10), it is expected that their effective anisotropy energy will grow, thus affecting the NFMR process in the composite. Furthermore, exchange coupling between the magnetically soft and magnetically hard phases is expected to improve the radar absorption properties of the RAC [90]. It was demonstrated [91] that the Ni_0.5_Zn_0.5_Fe_2_O_4_/SrFe_12_O_19_ composite filler can be considered as an efficient radar-absorbing component in a paraffin matrix (paraffin/filler ratio, 4:6). The filler was synthesized using the sol–gel method and by joint synthesis of the spinel phase and the hexaferrite phase from the same precursor (one-pot method). Strong exchange coupling between the spinel and hexaferrite phases occurs for a 1:3 ratio, and magnetic losses grow, as can be seen in the *μ*″(*f*) spectra. The maximum EMR attenuation is 47 dB at 6.2 GHz, with a 6.4 GHz working absorption bandwidth for a 4 mm thickness. The Ba(Zr–Ni)_0.6_Fe_10.8_O_19_/Fe_3_O_4_ composite filler synthesized by joint annealing in an argon gas atmosphere of separately obtained substituted hexaferrite and magnetite phases was studied [34]. The best radar absorption properties were obtained for an annealing temperature of 400 °C and a magnetically soft to magnetically hard phase ratio of 1:1. The authors noted that the working bandwidth of the RAC increased due to peak broadening in the *μ″*(*f*) spectrum of the composite.

Another method of producing efficient RAMs is to synthesize porous composites containing conductive and magnetic fillers. Along with dielectric and magnetic losses (NFMR and DWR), loss caused by multiple internal reflection in composite cavities can also be significant in these materials [92,93,94]. The properties of a radar-absorbing composite in which the filler is in the form of empty carbon microspheres coated with Fe_3_O_4_ magnetite nanoparticles having a hierarchic nanostructure were studied [92]. The synergy of magnetic and dielectric losses and multiple reflection loss caused scattering at the empty structures (the matrix/carbonized microsphere wall and the air/carbonized microsphere wall boundaries) provided for a maximum attenuation of 60.3 dB at a 10 wt. % filler content and a 3.72 mm thickness. Hierarchic structures consisting of porous CoFe_2_O_4_ and reduced graphene oxide were also synthesized [93]. The authors noted a synergetic improvement in radar absorption due to magnetic and dielectric losses (interfacial polarization) and eddy current loss in a 3D network of graphene sheets and multiple reflections in nanopores. A comparison between the radar absorption properties of the RAC in question is presented in Table 1.

Composite spinel ferrite RSM should contain either ferrite filler with a low electrical conductivity (magnetite, Mn, Cu containing spinel ferrites) or an additional conductive component. Then, one can achieve high attenuation coefficients (small skin layer thicknesses) and reduce the amplitude of incident EMR using low-thickness layers. However, excessive contents of conductive components can lead to high reflection coefficients. Then, a metal- or semiconductor-like shielding mechanism will be implemented in the composite RSM. Good radar-shielding composites are the above-discussed magnetic core/conductive shell structures [27,95,96,97,98]. The authors noted that magnetic core/conductive shell structures are EMW scattering centers, and the networks of interconnected particles are considered as EMW multiple reflection regions.

Obviously, to achieve the highest attenuation coefficient and radar absorption in materials, one should combine different EMR loss mechanisms. Aimed at achieving such a combination, a conductive polymer/polypyrrole/CoFe_2_O_4_/graphene composite was produced and shown to be an efficient RSM in the 8–12 GHz range with a low reflection coefficient [99]. Shielding in that composite occurred through radar absorption mechanisms. Porous composites with fillers of magnetic and conductive particles also perform very well as RSMs [100]. The introduction of carbon nanotubes and magnetite particles to a PVDF matrix followed by pore formation produces a porous composite with SE_T_ = 37 dB for a 2 mm thickness in the 14–20 GHz range. The use of magnetite as a filler in composite polymer RSM is an advantageous solution since magnetite can exhibit elevated electrical conductivity combined with magnetic properties (see above) and, hence, higher eddy current loss and interfacial polarization. For example, there are PVDF-matrix RSMs having the compositions PVDF/carbon nanotubes/Fe_3_O_4_ and PVDF/graphene sheets/Fe_3_O_4_ in which the shielding effectiveness is 35–37 dB at 18–26 Ghz for a 1.1 mm thickness [101]. The composites also exhibit good heat conductivity due to carbon material filling. Similar results were also obtained in other works [102]. A comparison between some polymer composite RSMs is presented in Table 2.

**Figure 5:**
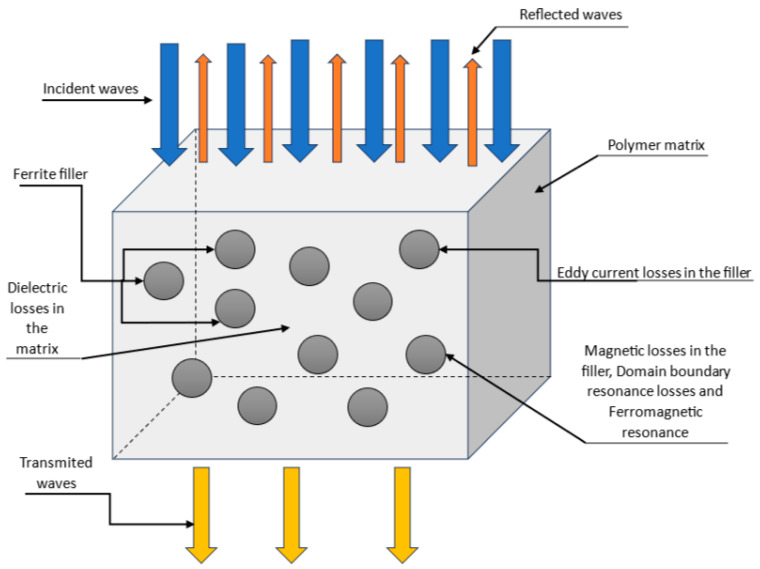
Scheme of the main mechanisms of absorption of electromagnetic waves in magnetic polymer composites with ferrite filler.

Figure 5 shows a diagram illustrating the main mechanisms of energy loss in electromagnetic waves when passing through magnetic polymer composites based on ferrites. These are the following mechanisms:

1. Reflection: part of the energy of electromagnetic waves is reflected.
2. Dielectric losses: part of the energy of electromagnetic waves is converted into heat due to dielectric losses in the polymer matrix.
3. Eddy current losses in ferrite filler.
4. Magnetic losses in ferrite filler: these are the energy losses of electromagnetic waves on the resonance of the domain boundaries and the ferromagnetic resonance in the ferrite filler.

It should be noted that all magnetic composites based on spinel ferrite fillers discussed in this review are non-flammable and solid. All of them are very technologically advanced from the point of view of implementing the application process on surfaces made of different materials. The issue of considering the mechanical properties of these composites is very important. However, this issue requires special research beyond the scope of this review. The same also applies to the problem of the influence of the particle size of the ferrite filler on the radio-absorbing characteristics of the considered composites.

## 5. Summary

EMR radar absorption in spinel ferrite polymer composites was reviewed. Those composites were shown to be promising as RAMs and RSMs due to the excellent magnetic and dielectric properties of spinel ferrites. The fundamentals of EMR interaction with materials, crystal structure and factors influencing the high-frequency behavior of spinel ferrites, main spinel ferrite synthesis methods and radar absorption and radar-shielding properties of polymer composites with spinel ferrites were discussed. Special attention was paid to the most efficient and proven methods of improving the radar absorption properties of spinel ferrite RAC. It was shown that reducing electrical conductivity and adding iron-substituting cations can significantly improve the radar absorption properties of spinel ferrites. The electrical conductivity of spinel ferrites is also a decisive factor controlling the frequency behavior of *R_l_* in two-component composites. Changes in the radar absorption spectra of ferrite–polymer composites in comparison with pure ferrites were explained based on the theoretical standpoints on the magneto-dynamic properties of ferrites. The improvement in the radar absorption properties of composites by combining magnetic fillers with conductive additives in the form of carbon derivatives, metals and polymers (magnetic core/shell structures) or by using combinations of magnetically soft and magnetically hard composites was discussed. It was stressed that device weight and dimensions can be improved by using porous composites with good radar absorption properties. The abovementioned regularities also hold true for radar-shielding spinel ferrite composites, in which shielding occurs via absorption mechanisms.

In their further research, the authors of this review plan to obtain and study the radio absorption characteristics of multilayer magnetic polymer composites, “polymer-ferrite”, as well as magnetic polymer composites with spherical ferrite particles coated with a metal film.

## Figures and Tables

**Table 1:** Comparison between radar absorption parameter *R_l_*(*f*) frequency behavior of ferrite–polymer composites.

Matrix	Filler	|*R_l_*(max)|, dB	*h*, mm	Δ*f* (−10 dB), GHz	*f*_0_, GHz	*C*, %	Ref.
PVA	Ni_0.32_Zn_0.68_Fe_2_O_4_	22.6	7	2.4	4.46	80 (wt. %)	[54]
PVA	Mn_0.58_Zn_0.26_Fe_0.16_Fe_2_O_4_ (100–200 μm)	22.26	6	2.3	4.42	40 (wt. %)	[64]
P(VDF-PFE), Grade F42	Mn_0.58_Zn_0.26_Fe_0.16_Fe_2_O_4_ (<45 μm)	24.2	5.9	2.49	5.37	20 (wt. %)	[63]
P(VDF-PFE), Grade F42	Mn_0.58_Zn_0.26_Fe_0.16_Fe_2_O_4_ (<45 μm)	68.79	20.2	0.16	0.06	80 (wt. %)	[63]
P(VDF-PFE), Grade F42	Mn_0.58_Zn_0.26_Fe_0.16_Fe_2_O_4_ (<45 μm)	49.67	16.2	0.52	0.98	40 (wt. %)	[63]
P(VDF-PFE), Grade F2M	Mn_0.58_Zn_0.26_Fe_0.16_Fe_2_O_4_ (45–100 μm)	42.55	6.5	2.52	4.07	40 (wt. %)	This work
Polystyrene	Mn_0.58_Zn_0.26_Fe_0.16_Fe_2_O_4_ (<45 μm)	29.75	6.8	2.4	3.57	60 (wt. %)	This work
P(VDF-PFE), Grade F2M	Ni_0.32_Zn_0.68_Fe_2_O_4_	27.5	6.9	3.37	5.37	80 (wt. %)	This work
P(VDF-PFE), Grade F2M	Li_0.33_Fe_2.29_Zn_0.21_Mn_0.17_O_4_	33.8	6	4.2	5.37	60 (wt. %)	[66]
P(VDF-PFE), Grade F2M	Li_0.33_Fe_2.29_Zn_0.21_Mn_0.17_O_4_	23.2	6	3.7	3.35	80 (wt. %)	[66]
Paraffin	Co_0.2_Ni_0.4_Zn_0.4_Fe_2_O_4_/graphene	53.5	3.1	4.8	10	40 (wt. %)	[77]
Paraffin	MnFe_2_O_4_/multiwalled carbon nanotubes	56.00	3	4 и более	11.41	40 (wt. %)	[80]
Paraffin	ZnFe_2_O_4_/multiwalled carbon nanotubes	42.06	3	4	11.05	40 (wt. %)	[80]
Paraffin	Ni_0.35_Co_0.15_Zn_0.5_La_0.02_Fe_1.98_O_4_	34	4	5.5	5.8	~85 (wt. %)	[71]
Wax	Ni_0.4_Zn_0.4_Mn_0.2_Fe_2_O_4_	29.56	4.5	2.5	8.7	80 (wt. %)	[72]
Wax	Ni_0.4_Zn_0.4_Cu_0.2_Fe_2_O_4_	35.02	4.5	2.5	8.6	80 (wt. %)	[72]
PVDF	Mn_0.8_Zn_0.2_Cu_0.2_Fe_1.8_O_4_	32	0.2	6	14	5 (wt. %)	[88]
Paraffin	Ni-Zn ferrite/polyaniline 1:1	44.23	2.25	1.65	12.1	25 (wt. %)	[49]
Paraffin	Ni-Zn ferrite/polyaniline 2:1	27.5	2	3	6	70 (wt. %)	[83]
Paraffin	Ni-Zn-Nd ferrite/polyaniline 100:25	37.4	4	4.9	8.3	37.5 (wt. %)	[84]
Paraffin	Ni_0.5_Zn_0.5_Fe_2_O_4_/SrFe_12_O_19_ 1:3	47	4	6.4	6.2	54 (wt. %)	[89]
Paraffin	Ba(Zr–Ni)_0.6_Fe_10.8_O_19_/Fe_3_O_4_ 1:1	43.08	1.9	6.88	14	-	[33]
Paraffin	CoFe_2_O_4_/rGO 1:2	67.58	2.1	6.3	13.5	10 (wt. %)	[81]
Paraffin	NiFe_2_O_4_/rGO	42	5	5.3	6.3	70 (wt. %)	[82]
Paraffin	CoFe_2_O_4_/CoFe alloy 4:6	58.22	1.45	4.16	12.96	80 (wt. %)	[75]
Wax	Fe_3_O_4_/carbonized empty nanospheres	60.3	3.72	6.4	9.5	10 (wt. %)	[91]
Paraffin	CoFe_2_O_4_ porous nanospheres/rGO 10:1	57.7	2.8	5.8	10.2	50 (wt. %)	[92]

**Table 2:** Comparison between shielding efficiencies of ferrite-containing polymer composite RAM.

Matrix	Filler	*SE_T_*, dB	*SE_R_*, dB	*SE_A_*, dB	Frequencies, GHz	*h*, mm	*C*, %	Ref.
PANI	Ni_0.6_Cd_0.4_Fe_2_O_4_	42	7	35	8–12	2.3	30 (wt. %)	[26]
PANI	Mn_0.2_Ni_0.4_Zn_0.4_Fe_2_O_4_	48.5	2.5	46	8–12	2.5	80 (wt. %)	[95]
PANI	Mg_0.6_Cu_0.4_Fe_2_O_4_	32.8	8.3	24.5	8–12	2.2	15 (wt. %)	[94]
PANI	Mn_0.5_Zn_0.5_Fe_2_O_4_	31	25	6	8–12	2	40(wt. %)	[96]
PANI	CoFe_2_O_4_	22	4	18	12.4–18	-	66 (wt. %)	[97]
PVDF	Mn-Zn ferrite	12–16	2	10–14	2–7	6	60 (wt. %)	This work
PVDF	Mn-Zn ferrite	15–35	2.5	12.5–32.5	2–7	6	80 (wt. %)	This work
Polypyrrole	CoFe_2_O_4_/graphene	38	1	37	8–12	2	44 (wt. %)	[98]
PVDF	Fe_3_O_4_/ПАНИ/carbon nanotubes	37	7	30	14–20	2	10 (wt. %)	[99]
PVDF	Fe_3_O_4_/carbon nanotubes (8% carbon material)	32.7	5	27.7	18–26.5	1.1	5.5 (wt. %)	[100]
PVDF	Fe_3_O_4_/graphene sheets (8% carbon material)	35.6	4	31.6	18–26.5	1.1	5.5 (wt. %)	[100]
PVDF	Fe_3_O_4_/soot	55.3	8.9	46.4	8–12	2	70 (wt. %)	[100]

## Data Availability

Data are contained within the article.
